# Cryoanesthesia with ethyl chloride spray versus 5% lidocaine gel in alleviating oral local anesthetic injection pain for buccal anaesthesia: A randomized clinical (controlled) trial

**DOI:** 10.34172/joddd.2023.37041

**Published:** 2023-04-03

**Authors:** Hira Abbasi, Faiza Ali, Hina Aslam, Muhammad Sharjeel Khan, Muhammad Waqas, Abhishek Lal

**Affiliations:** ^1^Department of Operative Dentistry and Endodontics, Altamash Institute of Dental Medicine, Karachi, Pakistan; ^2^Department of Dental Surgery, The Agha Khan University Hospital, Karachi, Pakistan; ^3^Department of Prosthodontics, Khyber College Of Dentistry, Peshawar, Pakistan; ^4^Graduate, Altamash Institute of Dental Medicine, Karachi, Pakistan; ^5^Department of Prosthodontics, Jinnah Medical and Dental College, Karachi, Pakistan

**Keywords:** Dental anxiety, Cryoanesthesia, Ethyl chloride, Topical lidocaine, Needle phobia

## Abstract

**Background.:**

Numbing the area of oral mucosa with cold application prior to administration of regional anesthesia has been widely used by various dentists in alleviating pain caused by needle prick. Cryoanesthesia using Endo-ice as topical anesthesia has been studied as a replacement to prevail the fallibility of topical anaesthetics. This study aimed to evaluate and compare effectiveness of ethyl chloride spray with 5% lidocaine gel in alleviating buccal anesthesia injection pain.

**Methods.:**

Total of 90 outpatients were randomly divided into 3 groups as follows: Group 1 – cryotherapy with ethyl chloride at the anesthetic site preceding before administration of local anesthesia; Group 2 – topical application of 5% LIDOCAINE GEL preceding before administration of local anesthesia; and group 3 – control that did not receive any topical agent preceding before administration of local anesthesia. Visual analogue scale (VAS) was used to document pain immediately after injection prick.

**Results.:**

About comparison of pain scores, significant difference was found between group 1 (ethyl chloride) and group 2 (topical lidocaine) patients (*P*=0.001). For group 1, about 15 (50%) patients suffered from mild pain, followed by 14 (46.67%) patients suffering from moderate pain. However, majority of the 21 (70%) patients in group 2 suffered from moderate pain. All the patients in group 3 suffered from severe pain.

**Conclusion.:**

Importance of alleviating fear of needle injection phobia amongst patients is of paramount importance. Ethyl chloride was found to be more effective than topical lidocaine in alleviating needle injection pain before administration of local anesthetic injection.

## Introduction

Local anesthesia is one of the most essential parts of almost all of dental procedures to ensure a painless experience for the patients and reduce their anxieties. Although different local anesthetic agents are available, lidocaine is one of the most commonly used drugs in the field of dentistry to provide painless dental treatment. Dental patients often ask for the application of topical agents that would make their initial prick of injection painless irrespective of age and gender.^1^

Fear of needle or needle phobia (also called as trypanophobia) is a well-known fear that disturbs many patients, especially during their first visit to the dentists. However, such fear of needles is not just limited to dentistry, but also can be an anxious experience for patients receiving any kind of medical care such as vaccinations and taking blood samples.^2^ Needle phobia can continue to disturb the patients during every dental visit of the patients if it is not properly managed by the dentists. Such fears if not addressed properly, can lead to avoidance behaviour by the patients toward the dental treatment.^3^ Dentists in their everyday practice come across patients with needle phobias, for which various techniques are used to cater to their needs.^4^ Whenever patients experience a painful dental procedure, that creates a vicious cycle of avoidance behaviour towards dental treatments which makes effective anesthesia a vital component of almost every dental procedure.

Lidocaine is one of the most commonly used anesthetic solutions in dentistry. Lidocaine is widely used primarily because of its low toxicity and potent anesthetic effects.^5^ Lidocaine comes in various concentrations and varieties for topical anesthesia and their respective effectiveness is well documented.^6^ Concentrations and varieties such as 2%, 4%, 5% solutions, 2% or 5% gel, and 10% spray are available for use. Various methods have been used preceding local anesthesia for numbing the site of needle insertion to make the procedure painless for the patient.^7-9^ With the advancement of various pharmacological methods for alleviating needle prick pain, there have been many studies on non-pharmacological methods as well.^10^

 Ethyl chloride is widely used among dentists in their everyday practice and is one of the standard methods to evaluate the baseline of pulp sensibility test. Ethyl chloride is widely used in the field of medicine as a pre-injection anesthetic and antiseptic properties for various medical procedures such as venipuncture and immunization. However, the use of ethyl chloride has not been consistent in dentistry.

A few studies report that cryotherapy or pre-cooling the area to receive local anesthetic has benefits over using topical local anesthetic agents are pain reduction, rapid onset, and patient compliance. However, very few studies have compared and evaluated the use of cryotherapy with other anesthetic modalities. Various pre-cooling agents have been used in multiple studies for the area to be anesthetized prior to the application of oral local anesthetic and have shown promising results.^11,12^

 To the best of our literature search, no study has been conducted to evaluate the effectiveness of pre-cooling the buccal mucosa with ethyl chloride compared to 5% topical lidocaine gel during buccal infiltration. Hence, we conducted this trial. In this study, we aimed to compare and evaluate the effectiveness of pre-cooling with ethyl chloride versus topical anesthesia on pain perception during buccal infiltration in adult patients receiving a local anesthetic injection.

## Materials and Methods

###  Study design and sample size

This randomized controlled trial was carried out between March and May 2022, in the Department of Oral & Maxillofacial Surgery, Sir Syed College of Medical Sciences for Girls, Pakistan. The ethical approval for this study was granted by the Ethical Review Committee of Sir Syed College of Medical Sciences for Girls, Pakistan. This study was carried out in accordance with the Declaration of Helsinki. This clinical trial was conducted according to the CONSORT guidelines and has been registered on the ClinicalTrials.gov site on 01/04/2022 with the number NCT05306470. The participants for this study were recruited by sampling strategy grouping via envelop method and participants were selected via non-probability convenience sampling method. The sample size for this study was calculated using OpenEpi software. Keeping the significance level at 5%, with power at 80%, and difference at 25%, the sample size was calculated to be 24 patients per group. (n = [(Z_α/2_ + Z_β_)^2^ × {(p_1_ (1 − p_1_)) + (p_2_ (1 − p_2_))}]/ (p_1_ – p_2_)^2^]).

###  Grouping and data collection

**Figure 1:**
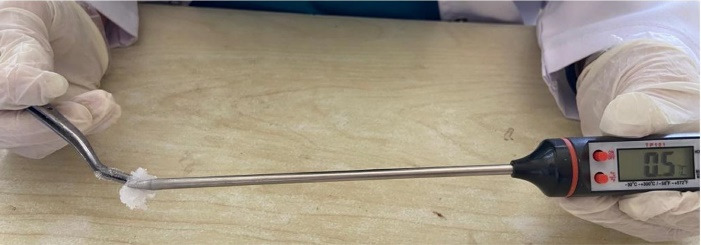
Measuring the temperature of Ethyl Chloride using a digital thermometer

**Figure 2:**
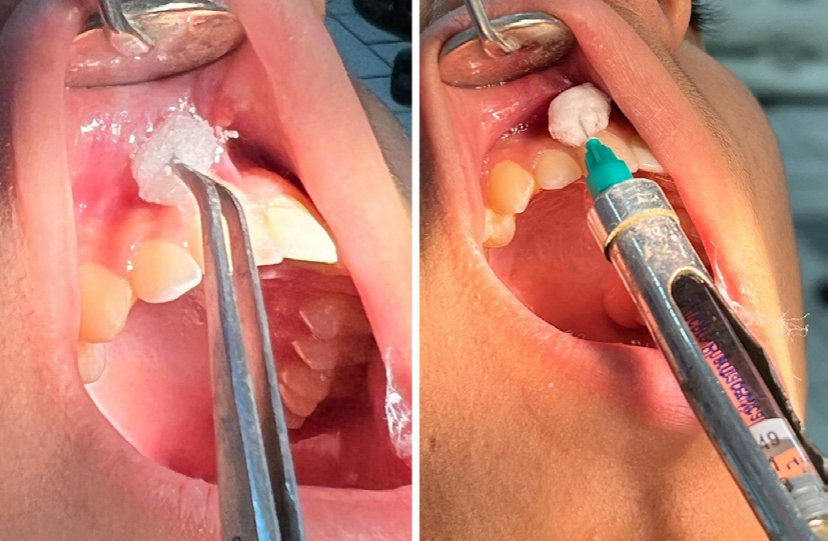
Application of ethyl chloride followed by local anesthesia

In this study, participants were randomly allocated to one of the three groups as follows: Group 1 − ethyl chloride; Group 2 − topical lidocaine; and group 3 − control. The principal investigator carried out the procedure of the clinical trial and subsequently assessed the pain scores of the patients. After adequate isolation and drying of the targeted area, one of the allocated techniques was performed. For Group 1 Cryotherapy with Ethyl chloride was used, where using a digital thermometer, the temperature of ethyl chloride was noted to range from -2°C to 0°C,^13^ as presented in Figure 1. A pea-sized cotton ball was sprayed with ethyl chloride and placed on the area to receive an injection for 30 seconds, later the local anesthetic solution (2% lidocaine with 1:80 000 adrenaline using a short 27-gauge needle (Septodont -Septoject Needles^TM^) on the targeted area was injected at slow speed for one minute using buccal nerve infiltrate anesthesia. After the administration of the injection patients were asked to rate their pain score according to visual analogue scale (VAS), as presented in Figure 2.

For group 2 application of 0.2ml of lidocaine 5% gel (Tehnodent, Desensetin^TM^) was performed with a cotton ball on the oral mucosa for 30 seconds and local anesthetic injection was administered after 5 minutes and pain score was documented using VAS after administration. In the control group, no local application for numbing the site was performed, only saline was used as placebo and the pain was evaluated using a visual analogue scale after administration of a local anesthetic. The procedure was followed by an buccal nerve infiltration injection by the principal investigator.

 For group 3, the patients did not receive any topical anesthetic agent and the pain scores of the patients was assessed using VAS after buccal nerve local anesthetic injection administration.

 About the inclusion criteria, The Participants with no medical history belonging to ASA classification 1, aged 20 to 40 years, no gender restriction, no history of medication, patients not allergic to lidocaine, and patients requiring buccal infiltration for their treatment were included in this study.

 Ibuprofen 800 mg was given to the patient one hour before the treatment in order to address in pain before infiltration anesthesia.

 About the exclusion criteria, however, the patients who failed to fulfill the inclusion criteria were eliminated from the study, including those unamenable to treatment and who did not give their consent to participate in the study.

###  Measurement of pain score

**Figure 3:**
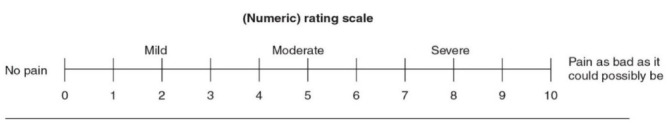
Numeric rating scale

To measure the pain scores of the patients, a numeric rating scale was used. A numeric pain scale was used, according to which the pain was categorized as follows: 0 = No pain and 10 = Severe pain.^14^ It was further subdivided into categories as follows: 0 = No pain, 1-3 = Mild pain, 4-6 = Moderate pain, and 7-10 = Severe pain, as presented in Figure 3. As the score of the pain increases, so does the severity with which the patient is experiencing the pain.

###  Informed consent

 Informed consent was sought from each of the participants before conducting the study. Participants were informed about the purpose and nature of the study through the information and consent form. Participants who agreed to it were asked to sign a consent form. Participants’ identity was kept confidential throughout this study. However, their responses and results of this study were shared without mentioning their names.

###  Data analysis

For the data analysis, SPSS Statistical Software (version 25, Armonk, NY: IBM Corp.) was used. The mean and standard deviation was calculated for the demographic variables. One-way ANOVA and post hoc analysis was used to compare the mean pain score amongst the groups. Multiple linear regression analysis was used to compare the effect of age and gender on the pain scores. A *P* value of ≤ 0.05 was considered to be as statistically significant.

## Results

**Figure 4:**
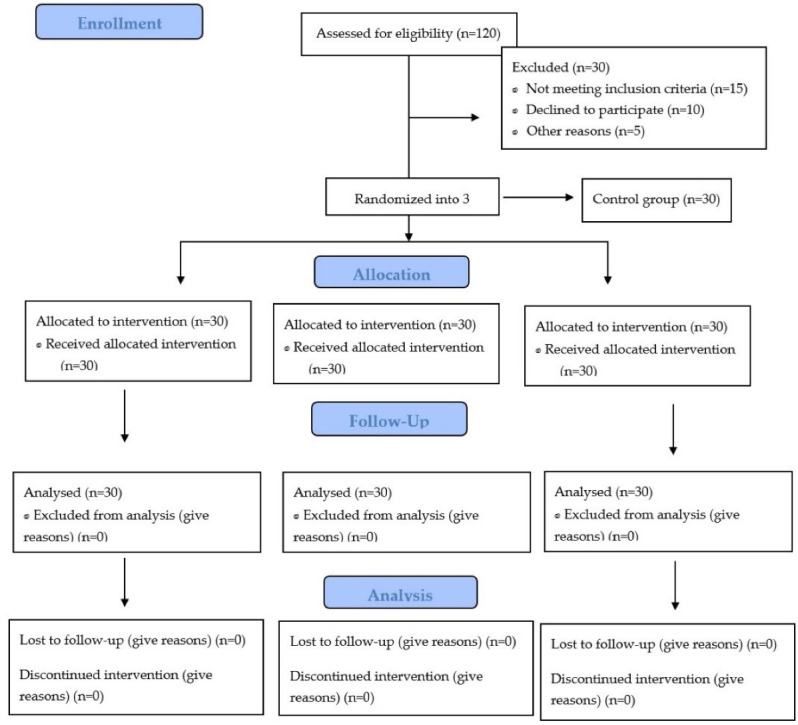
CONSORT Flow diagram (n = 90)

In this randomized controlled trial, a total of 90 patients participated, as presented in the CONSORT flow diagram (Figure 4). The mean age of the patients in each group was as follows: Group 1 28.7 ± 5.5, Group 2 30 ± 6.25, and Group 3 28.5 ± 5.4. The distribution of males and females in each group was as follows: Group 1: 16 and 14, group 2: 13 and 17, and group 3: 18 and 12, as presented in Table 1.

**Table 1:** Demographic characteristics of the patients amongst different groups (n = 90)

**Variables **	**Group 1: Ethyl Chloride (n=30)**	**Group 2: Lidocaine (n=30)**	**Group 3: Control (n=30)**
Age (y)			
Mean ± SD	28.7 ± 5.5	30 ± 6.25	28.5 ± 5.4
Gender			
Male	16 (53.33%)	13 (43.33%)	18 (60%)
Female	14 (46.67%)	17 (56.67%)	12 (40%)

 For group 1, the treatment required by the patients were as follows: 20 (66.67%) root canal treatment, 10 (33.33%) extraction of teeth. For group 2, the treatment required by the patients were as follows: 24 (80%) root canal treatment, 6 (20%) extraction of teeth, and For group 3, the treatment required by the patients were as follows: 21 (70%) root canal treatment, 7 (23.33) extraction of teeth, and 2 (6.67%) deep caries removal. Root canal treatment was performed on patients suffering from irreversible pulpitis, unsalvageable teeth were extraction, teeth with reversible pulpitis underwent filling treatment.

**Figure 5:**
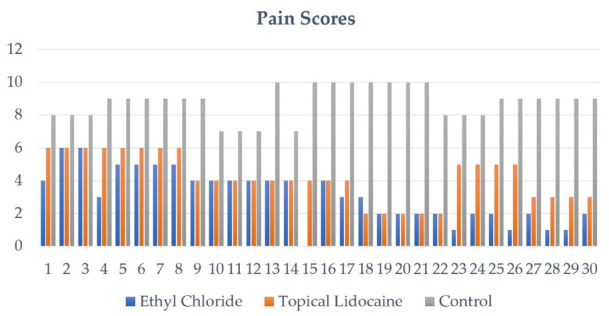
Pain scores of patients amongst the three groups (n = 90)

The mean scores of patients belonging to each group was as follows: Group 1: 3.10 ± 1.605, group 2: 4.2 ± 1.42, and group 3: 8.8 ± 0.99. Regarding the pain scores of patients in group 1 who were given ethyl chloride, about 15 (50%) patients suffered from mild pain, followed by 14 (46.67%) patients suffering from moderate pain, and lastly only 1 (3.33%) patient did not suffer from pain upon administration of local anesthesia, as presented in Figure 5. Secondly, regarding the pain scores of patients in group 2 who were given topical lidocaine, the majority of the 21 (70%) patients suffered from moderate pain, followed by 9 (30%) patients who suffered from mild pain with none of the patients suffering from severe or no pain. Furthermore, among patients belonging to group 3 control, all of the patients reported suffering from severe pain, as presented in Table 2.

**Table 2:** Distribution of pain scores in proportions after the use of Ethyl chloride, topical lidocaine, and in control group for reduction of injection pain

**Pain scores**	**Group 1: Ethyl chloride (n=30)**	**Group 2: Lidocaine (n=30)**	**Group 3: Control (n=30)**
Score 0	1 (3.3%)	0 (0%)	0 (0%)
Score 1	4 (13.3%)	0 (0%)	0 (0%)
Score 2	8 (26.7%)	5 (16.7%)	0 (0%)
Score 3	3 (10%)	4 (13.33%)	0 (0%)
Score 4	8 (26.7%)	9 (30.0%)	0 (0%)
Score 5	4 13.33%)	4 13.33%)	0 (0%)
Score 6	2 (6.67%)	8 (26.67%)	0 (0%)
Score 7	0 (0%)	0 (0%)	4 (13.33%)
Score 8	0 (0%)	0 (0%)	6 (20%)
Score 9	0 (0%)	0 (0%)	12 (40%)
Score 10	0 (0%)	0 (0%)	8 (26.67)

Regarding the comparison of mean value of pain scores using one-way ANOVA, a significant relationship was noted between the three group (*P* = 0.001). Using post hoc analysis, a significant relation was noted between groups 1 and 3 (*P* ≤ 0.001) and groups 2 and 3 (*P* ≤ 0.001, as presented in Table 3.

**Table 3:** Intergroup comparison amongst the patients of three groups (n = 90)

**Groups**	**Agents**	**Mean difference**	**Standard Error**	**95% Confidence interval**		***P*** ** value**
				**Lower bound**	**Upper bound**	
Group 1: Ethyl Chloride	Lidocaine	-1.10	0.35	-1.94	-0.26	0.193
	Control	-5.70	0.35	-6.54	-4.86	0.001
Group 2: Lidocaine	Ethyl chloride	1.10	0.35	0.26	1.94	0.193
	Control	-4.60	0.35	-5.44	-3.75	0.001
Group 3: Control	Ethyl chloride	5.70	0.35	4.85	6.54	0.001
	Lidocaine	4.60	0.35	3.75	5.44	0.001

The comparison of age and gender with the pain scores of the patients was analyzed using multiple linear regression. Regarding the comparison of gender with pain scores, a statistically significant difference was noted (*P* = 0.003). However, no statistically significant difference was noted between age and pain scores (*P* = 0.913). as presented in Table 4.

**Table 4:** Comparison of age and gender with pain scores of the patients (n = 90)

**Variables**	**Unstandardized coefficients**		**Standardized coefficients**	***t***	***P*** ** value**
	**B**	**Standard Error**	**Beta**		
Age	0.006	0.053	0.012	0.109	0.913
Gender	-1.839	0.609	-0.327	-3.01	0.003

## Discussion

 Fear of administration of local anesthesia, also known as needle phobia, is a well-known and major problem faced by patients as well as dentists. For the optimal success of most of the dental procedures, adequate pain control is of paramount importance as a pain-free environment decreases the stress as well as anxiety of the patients. Lidocaine is one of the most commonly and widely used local anaesthetic agents in dentistry in the form of block and infiltrate anesthesia.

Different studies have evaluated and found different methods to alleviate needle prick pain of the patients when buccal anaesthesia is administered, where topical anesthesia is one of the commonly used modalities.^15,16^ Firstly, in our study, we used ethyl chloride in group 1 patients to assess their levels of pain upon needle prick for buccal anesthesia. For group 1 patients, about 15 (50%) patients suffered from mild pain, followed by 14 (46.67%) patients suffering from moderate pain. Similar findings have been reported in a study by Chilakamuri et al, where when patients were given a pre-cooling agent for before local anesthesia, they reported a significant reduction in their pain scores as compared to topical anesthesia.^17^ However, one study does suggest that ethyl chloride increased the level of patient’s pain perception as compared to sampling procedure transabdominal chronic villus sampling.^18^ Hence, the application of pre-cooling in patients might vary from procedure to procedure. Moreover, one study has also used a fast-acting refrigerant which was found to provide similar pain relief as compared to topical anesthetic gel.^19^ A further benefit associated with using ice is associated with decreasing the discomfort of the patients upon being administered needle injections.^20^

Secondly, in our study, we used topical lidocaine in patients before administration of local anesthesia to assess their pain scores. We, in our study, found that the majority of the patients suffered from moderate pain when local anesthesia was administered to them. Such results correspond with a study by Nusstein and Beck, where about 30% of the patients suffered from moderate to severe pain upon being given local anesthesia.^21^ Moreover, one study also concluded that pre-cooling was slightly better in pain relief of the patients as compared to using topical lidocaine.^22^ However, one study suggests that the use of topical lidocaine resulted in alleviating the pain of patients prior to local anesthesia administration.^5^ Since the level of pain perception varies from individual to individual, there might be a difference in pain scores despite any pain alleviating modality being used for the patients.

Besides, ethyl chloride and topical lidocaine, when the patients were not provided with any pain alleviating modality as in group 3, all of the patients in our study reported suffering from severe injection pain. Moreover, in our study, females experienced greater severity of pain upon injection administration as compared to males. Similar results have been reported in different studies that do conclude females suffer from greater levels of pain.^23,24^ Furthermore, in our study, there was no significant relation of pain scores with age. However, literature does suggest that young adults and paediatric patients tend to suffer from greater levels of pain.^25^

Different pain alleviating modalities have been used in literature other than ethyl chloride and topical lidocaine. Such modalities include benzocaine gel, and clove-papaya based gel with encouraging success in alleviating the pain of the patients noted.^26^ Moreover, the technique of administration of local anesthesia has also been known to affect the perception of pain by the patients. The use of small needles of local anesthesia along with warming the solution of local anesthetic has shown to decrease the injection pain of the patients.^27^ Future clinical studies should focus on evaluating and comparing the use of ethyl chloride with pain alleviating modalities in order to further determine its effectiveness. There is always room for improvement to improve the field of painless dentistry.

 In our study, it was concluded that both ethyl chloride and topical lidocaine successfully decreases the pain scores of the patients, with ethyl chloride having greater effectiveness as compared to topical lidocaine. Despite the strengths of this study such as adequately measuring pain scores of the patients and techniques used to alleviate injection pain of the patients, we were met with some limitations. Firstly, we had a small sample size of patients. Lastly, unequal distribution of males and females in all of the groups might have affected the perception of pain scores amongst the participants.

## Conclusion

 Effective management of injection pain prior to local anesthesia administration is of paramount importance, especially to patients who suffer from needle phobia. Cryotherapy with the help of ethyl chloride significantly alleviates pain perceived by patients receiving an injection of local anesthesia. Therefore, ethyl chloride can be used as a pain alleviating modality for patients.

## Acknowledgments

 The authors would like to thank all the participants who participated in this study.

## Competing Interests

 The authors declare there are no conflict of interest.

## Data Availability Statement

 The datasets generated during and/or analysed during the current study are available from the corresponding author on reasonable request.

## Ethical Approval

 All the participants in this study agreed to the use of their data by providing verbal and written informed consent. All the experiment protocols and consent were approved by the Ethics committee of Sir Syed College of Medical Sciences for Girls (sscms/college/principal/(dental)/2022/071). All the clinical investigations were conducted in accordance with the guidelines of the Declaration of Helsinki.

## Funding

 This study did not receive any funding.
